# A botanic garden as a tool to combine public perception of nature and life-science investigations on native/exotic plants interactions with local pollinators

**DOI:** 10.1371/journal.pone.0228965

**Published:** 2020-02-20

**Authors:** Manuela Giovanetti, Claudia Giuliani, Samuel Boff, Gelsomina Fico, Daniela Lupi

**Affiliations:** 1 Centre for Ecology, Evolution and Environmental Changes, University of Lisbon, Lisbon, Portugal; 2 Department of Pharmaceutical Sciences, University of Milan, Milano, Italy; 3 Department of Pharmaceutical Sciences, Ghirardi Botanic Garden, University of Milan, Milano, Italy; 4 Department of Food, Environmental and Nutritional Sciences, University of Milan, Milano, Italy; Instituto Federal de Educacao Ciencia e Tecnologia Goiano - Campus Urutai, BRAZIL

## Abstract

Life-sciences are pointing towards an alarming worldwide pollinator decline. This decline proceeds along with overall biodiversity losses, even in the context of urban landscapes and human welfare. At the same time, social-sciences are arguing an increased distance from nature, experienced by citizens. The strong connection between the public good and pollinator sustainability, even in urban areas, is well-documented. However, usually basic and applied life-sciences tend to underestimate public perception of nature, which is better tackled by the fields of social-sciences. Therefore, more efforts are needed to link scientific questions and public ‘perception’ of nature. We designed a transversal project where research questions directly confront public concerns: i.e., even while addressing scientific knowledge gaps, our questions directly arise from public concerns. Social studies highlighted that appreciation of (exotic) plants is related to the impact they may have on the surrounding natural environment: therefore, we investigated links of native and exotic flowers to local pollinators. Other studies highlighted that scientific results need to link to everyday individual experience: therefore, we investigated pollination modes of the renown *Salvia*, native and exotic, largely used in cuisine and gardening. The botanic garden was the promoter of scientific questions addressed by the public, and also collated the results in a travelling exhibition. The exhibition, together with a dedicated catalogue, were especially designed to enlighten the wide public on the relationships that plants, native and exotic alike, establish with the surrounding world.

## Introduction

The public perception of nature has changed through human history, more recently facing two main drivers. These are pushing in opposite directions: urbanization, that heavily reduces daily contacts with plants or animals, and science, which constantly enlarges knowledge and gives new insights on sustainable living choices to preserve nature. Still no consensus exists on how to measure the importance of natural resources, the services provided by natural ecosystems, the connections of nature-enriched environments with health and quality of lifetime [1,2,3,4]. The natural world is often perceived as a “surrounding” environment, to which most of us is not directly connected. Instead, our own survival strongly depends on the connections with it [5]. Plants are of enormous importance as providers of oxygen, food, and as sources of pharmaceutical products that we need to contrast illnesses. They sustain human well-being and are food sources and shelters to animals interacting with them.

Pollinators, especially bees, are also of enormous importance: they provide one of the most important ecosystem services [6,7]. Pollinators are the vectors that plants use to produce new generations through the processes of fertilisation, fruit and seed formation [8,9,10]. Currently there is a need to keep pollinators at the spotlight, since large diversity of the food (e.g. fruits and seeds) consumed by human, wild and domesticated animals, and even pets, rely on pollination to be produced [11,12,13].

How people react to plants and nature-related concepts can however result surprising. An example is the concept of native/exotic species. Hoyle and colleagues [14] investigated the public perception of non-native plants in gardens highlighting key factors that are actively driving acceptance or rejection of a given landscape by the public. As expected, the aesthetically pleasing appearance was one of them: beautiful flowers are accepted and planted in gardens, independently from their country of origin. However, it also turned out that potential incompatibility with native wildlife plays a role in granting acceptance of non-native species: knowledge on how plants influence the local environment may change people’s mind.

Urban gardens lately received an increasing attention as repository of intrinsic values: both, for people well-being [15,16] and for the ecological services they sustain [17,18]. Back in 2009, Frankie and colleagues [19] disseminated the results of a large study involving gardens in seven Californian cities: they underlined the intrinsic value of gardening as habitats for native bees. Again in 2009, Pawelek and colleagues [20] highlighted how it was possible even to increase local pollinators in urban gardens by choosing different plants and garden designs. Almost ten years after, Burr and colleagues [21] provided useful conservation direction for yards in the USA, melting data on insect pollinator populations and social and cultural drivers influencing people choices. Similar studies, that provide information on compatibility between ornamentals and pollinator sustainability [i.e. 22,23,24], perfectly match the increased apprehension about dramatic pollinator's losses [25,26,27,28] that recently also reached the wide public [29].

Botanic gardens (BG) are special places where merging exotic plant species and their relationship to local wildlife, finally guiding human perception on how they interact and what may result by hosting exotic species [30,31]. BGs are special since the plants they host are not casually selected: they have been planted and catalogued according to precise criteria: an example is the status of each species (e.g. exotic, rare, endangered, with great conservation value, etc.) [32]. Moreover, they have a strong historical value that the public can appreciate. These gardens appeared in the Middle Age in convents or monasteries: they collected medicinal plants, indigenous or exotic, employed for the care of various sicknesses. In the Renaissance, they became places for the collection, cultivation and study of plants with healing properties (the first in Pisa, Italy, in 1543/44) [33]. During the eighteenth century, the period of the great explorations, BGs hosted the exotic species coming from newly discovered countries with the aim of experimenting their ornamental and economic potential. Currently, in the context of biodiversity losses, BGs have assumed a new role as repositories for the conservation of the plant biological diversity at global level [34,35]. In addition, a fundamental mission of BGs is linking public direct experience to the perception of the importance of natural systems, greeting citizens as pleasure and relaxing sites [36] while concurrently acting as “open-air museums”.

We planned a transversal project, where the public perspective drove the focus of scientific research plans: 1) to address the recent findings of granting acceptance of non-native species when connected to positive compatibilities with native wildlife, we investigated exotic and native species in relation to pollinators visits. In this case, we expected *BG* to act *as a plant-pollinator network repository*: considering their urban location and the abundance of plant species with different flowering time, BGs may constantly sustain local population of pollinators. During two following years we monitored bee visits and analysed differences and similarities comparing the respective networks of exotic and native plant species, with the aim of verifying suitability of exotic species as food sources for local bees; 2) to sustain the need of deepening the connection of nature with everyday human life [37,38,39], we addressed to sages, renown species traditionally used for cooking and frequently planted in private and common gardens. We performed a comparative study using the *BG as an open-air laboratory*: it was a perfect location, since *Salvia* species of different geographic origin acclimatised during a long period and, notwithstanding their home range, potentially share the native pollinators assemblage. We measured flower characteristics of five *Salvia* species, recorded and identified pollinators visiting them, compared pollinators assemblages and pollinators frequencies among specie. Our aim was selecting the mostly-liked by local pollinators giving possible suggestions on the best-suited species for gardening activities; 3) with the aim of strengthen the link between scientific findings and society [40,20] and promote understanding and conservation effort, *BG* was *the interactive learning promoter* of a national travelling exhibition titled “*Seduzione / repulsione*: *quello che le piante non dicono*”(*Seduction Repulsion–what plants do not say*) associated to an illustrated catalogue [41]. The BG promoted the exhibition and the catalogue by implementing their setting-up with the involvement of local and national stakeholders, but was also actively hosting and spreading the content of the exhibition. In these deliverables, plants were presented not only for the aesthetic appeal of their flowers: they were illustrated according to the interactions they establish with other organisms, the evolutionary paths that drove them to develop given strategies, finally underlining how these strategies are not different from those also employed in human activities.

## Materials and methods

### Study site

This study was performed at the Ghirardi Botanic Garden (GBG) of Toscolano Maderno (Italy), on the western shore of the Lake of Garda at 86 m asl. The Ghirardi Botanic Garden (GBG) of Toscolano Maderno (Italy) granted permits to carry out field research in its premises. The town has a municipal land area of 56.73 km^2^, of which 0.78 urbanized (urbanized surface incidence = 13.77%). In 2017, the population census reported 7969 actual residents (population density = 135.41 residents/km^2^) plus about 7000 transient inhabitants/tourists temporarily present in camping, hotels or other accommodation facilities during the spring and the summer. The climate is mild, generally warm and classified by the Köppen-Geiger system as continental temperate, with hot summer (Cfa). About 844 mm of precipitation falls annually; hottest months are July and August with respective mean daily maximum temperatures of 28°C and 29°C [42]. GBG, established in 1964 as an experimental botanic station under the direction of Prof. Giordano Emilio Ghirardi, extends over a surface of about 10000 m^2^ and, currently, the preserved plant heritage includes more than 400 *taxa* from all the regions of the world. GBG has a long history of hosting medicinal species of different origin, in relation to the favourable microclimate of the site which facilitated the acclimatization of the introduced plants. The primary purpose was the cultivation and preservation of officinal species, mainly with cardiotonic and antitumor properties. It is worth mentioning the Chinese *Camptotheca acuminata* Decne. (Cornaceae), whose seeds were sent to GBG for a presumed anticancer activity, finally documented only by recent studies. Since 2002 it is part of the non-profit network “Rete degli Orti Botanici della Lombardia” (“Network of Botanic Gardens of Lombardy”).

### GBG as a plant-pollinator network repository

Plant data collection started with an accurate screening, including the earliest checklists up to the most recent contributions and reports [43]. To match pollinator visits to plants, we recorded visits along linear transects every two weeks during two following seasons (March-September 2016 & 2017; n = 22 transects). Due to the numerous plant species and their different flowering durations, repeated walking-transects are the most suited method to detect if frequency of visits may be considered occasional (a single visit is recorded) or if some kind of constancy is observed. Along the transects, 244 plant species were present, belonging to 63 families (S1 Table). However, for further analyses only species showing attractiveness towards flower visitors were considered. Bee individuals were recognised at sight till the deepest possible level and recorded only once when visiting a species, even in the case of paying multiple visits at flowers simultaneously present on the plant. We also took photographic evidence of bee visits, double-checking with final bee identification list, and collected specimens. We grouped visited plant species according to their provenance: native, if originally of Mediterranean or continental Europe; exotic, if from other continents or islands. Further, they were grouped according to Pellissier and colleagues' [44] classification of flower characteristics: wind, disk, funnel, bilabiate, tube, head, brush (S1 Table). These floral morphologies vary as for availability and accessibility of the floral resources, pollen and nectar; therefore, they imply different attraction potential towards diverse groups of pollinators. To depict preferences of bees in flowers with different morphologies, we constructed a network visualisation using R 2.14.0 (R Core Team, 2013), keeping exotic and native species separated.

### GBG as an open-air laboratory

GBG hosts numerous species of the mint family (Lamiaceae). We selected sages, our target being five *Salvia* L. species differing by native range, flower characteristics and pollination ecology. Indeed, the genus has some peculiarities regarding pollination strategies, either carried on by bees or by birds, and compound emissions [45,46,47]. Two species were native to Europe (*Salvia pratensis* L. and *Salvia verticillata* L.) and three exotic, of central-south American origin (*Salvia blepharophylla* Brandegee ex Epling, *Salvia greggii* A.Gray and *Salvia uliginosa* Benth.). These species were not only divergent as geographical origin: they also originally co-evolved with different pollinators: insects (mainly bees) for *S*. *uliginosa*, *S*. *pratensis and S*. *verticillata*, and birds (mainly hummingbirds) for *S*. *blepharophylla* and *S*. *greggii*. In temperate areas as the one where the GBG is located, bird-pollination is not an option. Our aim was verifying if all species equally sustain native pollinators, being rich in nectar and often planted in gardens as ornamentals or for culinary purposes. To investigate pollinators on them, we first performed a literature search on the five species, acknowledging only citations referring to observed visits. We revised the first 50 citations obtained as Google Scholar output under comparable keywords (pollinator / *Salvia* 'name of species'). In the genus *Salvia*, as in the whole family Lamiaceae, the flower is bilabiate and characterized by the typical staminal lever, a mechanism helping in a successful pollen deposition on the pollinator’s body. For testing the morphological variability at flower level, 20 randomly-selected fully-opened flowers per species were compared for what concerns the inflorescence type, the floral colour and the total length of the corolla. We measured the last parameter using a digital calliper and a stereomicroscope and evaluated three different size class: short (<1.5 cm), medium (1.5–3.0 cm), long (>3.0 cm). We directly recorded flower visitors on each species on sunny days, between 8:00 and 14:00 (solar hour). Patch records [48] regarded bee observation, were repeated along the day and lasted 10 minutes (221 patch records in total). Data refers to 10 days, from May to September 2016 fortnightly. Each bee approaching the flower was recorded and classified as explained for the plant-pollinator network: each individual accounted for a single visit, notwithstanding the amount of visited flowers.

### GBG as the interactive learning promoter

For this project, the GBG was the promoter, and directly involved, in the setting-up of a travelling exhibition and a printed catalogue. Information on plants and their communication media with other organisms were selected: texts, photographic material and drawings were all employed for the final editing. Multi-stakeholder’s meetings involving scientist, artists and botanic garden managers were planned in order to decide how to settle up the mobile exhibition. Selection of material to be included in the panels and how to relate it with plants in the garden was also the result of open discussions. Mobile panels were printed and exposed in the GBG greenhouse (S2 Table). Some plant species hosted in the garden where selected to recall the information of the panels and flagged accordingly: this way, the information read on panels was transferred to a direct experience while visiting the garden and its plant content. The exhibition was displayed across Italy thanks to the involvement of the Network of Botanic Gardens of Lombardy, and other stakeholders as local municipalities. The catalogue [41] was reflecting the panel order, reproducing part of the same content, comprehensive of texts, photographic material and drawings. A colloquial language was mainly applied to explain context of concepts; however, scientific terminology was also employed and fully explained. Moreover, to increase empathy we highlighted those strategies adopted by men for communication efforts during artistic and social performances that resemble the ones adopted by plants.

## Results

### GBG as a plant-pollinator network repository

**Fig 1:**
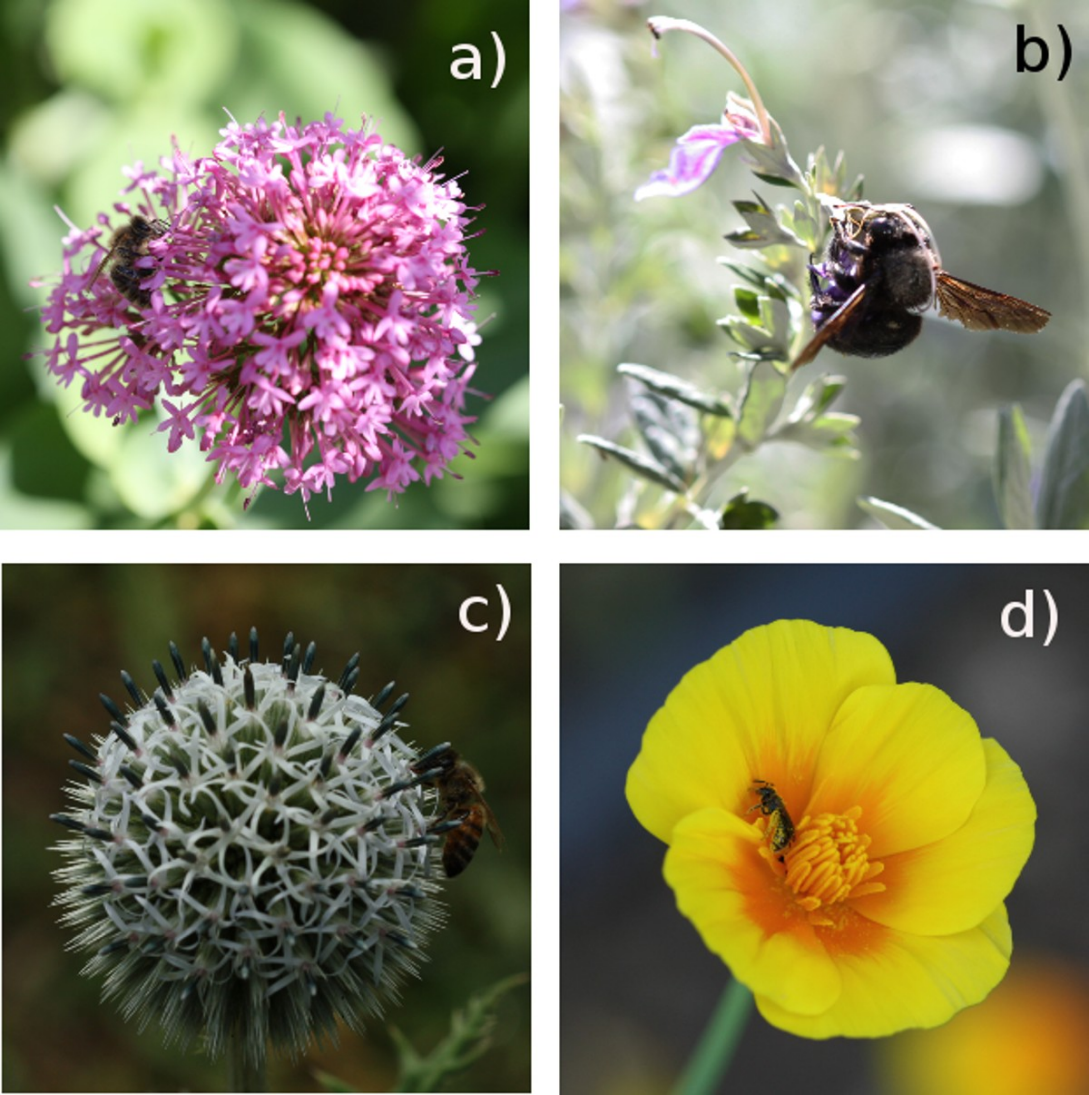
Examples of plants hosted in the GBG and native pollinators visiting them. a) *Centranthus ruber* (L.) DC (native, funnel) and *Apis mellifera* L; b) *Teucrium fruticans* L. (native, bilabiate) and *Xylocopa violacea* (L.); c) *Echinops sphaerocephalus* L. (exotic, head) and *Apis mellifera*; d) *Eschscholzia californica* L. (exotic, disk) and *Lasioglossum* sp.

The linear transects contained 244 plant species, out of which 140 (57.4%) received at least a visit by a local pollinator (see S1 Table). Abundance of native and exotic species along the transects was very similar: 44.3% of exotic species and 55.7% of native ones. While walking along the transects, we recorded 517 bee visits on flowers, including those made by unidentified bees. Exotic and native plant species experienced similar amounts of occasional (26 and 23, respectively) or multiple visits (36 and 55, respectively), no difference emerging between natives and exotics (Fishers's exact test: p = 0.154). The majority of visited species (61.1%) experienced more than one visit, up to a maximum of seventeen. Also combining data and looking for overall number of visits received by all exotic or all native species, no difference emerged (*t* = 1.7242, df = 130, p = 0.0871). Average number of visits would be 3.05 visit/exotic- and 3.97 visit/native plant species. Notwithstanding their origin, native and exotic plant species obviously showed convergence of flower characteristics (Fig 1). When considering flower morphology, among the visited species we observed that brush blossom was not represented at all, while tube and wind flower morphotypes were poorly represented: 5 records of visits in total, native and exotic combined. The other four categories (bilabiate, disk, head and funnel) were all similarly visited: 30.9% of visits to bilabiate, 20.8% of disk, 17.2% of head and 30.1% of funnel. Minor differences in trends were shown by the two groups. Among the exotic species, number of visits where in decrescent order for head, disk and funnel blossoms, while for native species highest visits were on bilabiate, funnel and disk blossoms. No preferences emerged in the number of visits recorded, when addressing flower morphotype and origin of the species, not even for the two most represented groups (for bilabiate blossom, Fishers's exact test: p = 0.1094; for funnel, Fishers's exact test: p = 0.1517).

**Fig 2:**
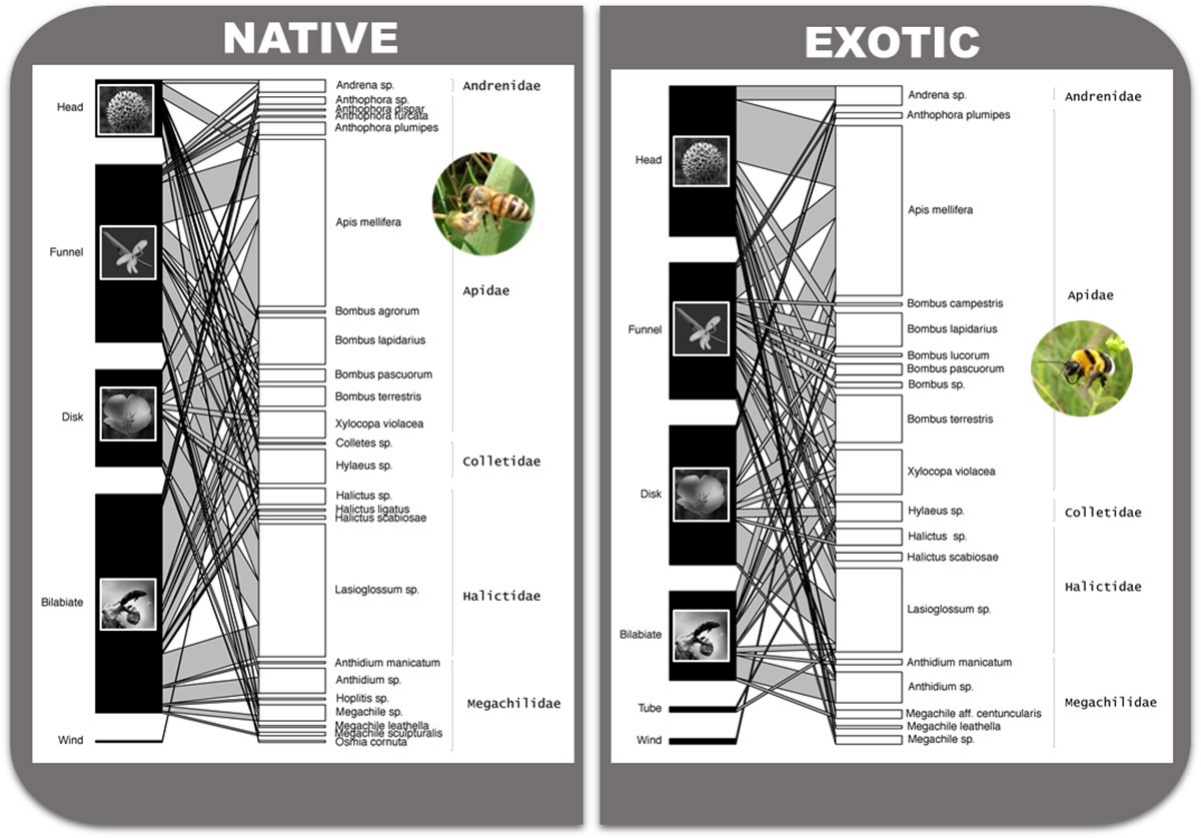
**Native (n = 73; A) and exotic (n = 59; B) plant–pollinator networks.** Bars on the left side represent plants, grouped according to flower morphologies; bars on the right side represent bees, at species level when possible. An indication of the family to which bees belong is reported on the extreme right side of each plot, after bee (genus/species) name. Linkage width indicates the number of individuals of that bee species paying visits to plants with given flower morphology. The length of the bars for plant species represents the frequency of species with a given flower morphology. The length of the bars for the bee species represents the total number of individuals recorded for that species on all plant species combined.

Only a few bees could not be ascribed to any of the following families: Apidae, Andrenidae, Colletidae, Megachilidae and Halictidae. The five families were differently represented, as number of visits (S1 Table). The highest frequency of bee visits was due to the family Apidae (56.5%), shared between the *Apis mellifera* L. (honeybee) and *Bombus* Latreille species (bumblebees). The second family in order of importance was that of Halictidae (25.2%). The remaining 18.3% was due to Andrenidae, Megachilidae and Colletidae. Fig 2 represents the visualisation of networks between native or exotic plants and local pollinators. The network also highlights the relative abundance and visitation rates of plants grouped accordingly to the flower morphotype they were sharing.

### GBG as an open-air laboratory

Results of the literature search (Table 1) highlights that the most frequent pollinators observed on the five species of sages were the honeybee (*Apis mellifera*) and the bumblebees (*Bombus* spp., various species). *Xylocopa* spp. also seemed quite attracted to sages, being previously recorded on three out of the five *Salvia* species.

**Table 1:** Selected existing literature on the five *Salvia* species considered in this study. We are reporting studies indicating pollinator visits actually observed by authors.

*Salvia* species	Study location	Observed pollinators	Reference list
*S*. *greggii*	USA, California	*Calypte costae* (Bourcier) (hummingbird), *Apis mellifera* L. and *Xylocopa* spp.	[19,49]
*S*. *blepharophylla*	-	-	-
*S*. *verticillata*	Poland	*Apis mellifera*, *Bombus* spp., *Bombus terrestris* L., *B*. *lucorum* L., solitary bees, flies, butterflies	[50,51,52]
	Italy	*Bombus* spp., *Xylocopa violacea* (L.), *A*. *mellifera*, *Anthidium* sp., *Lasioglossum* sp., *Hyleus* sp., *Andrena sp*.	[47]
	Iran	*Apis mellifera*	[53]
*S*. *uliginosa*	USA, California	*Xylocopa* sp., spring bees	[20]
*S*. *pratensis*	UK	*Bombus pascuorum* (Scopoli), *B*. *Hortorum* (L.), *B*. *Lapidarius* (L.), *Apis mellifera*, hoverflies	[54]

**Fig 3:**
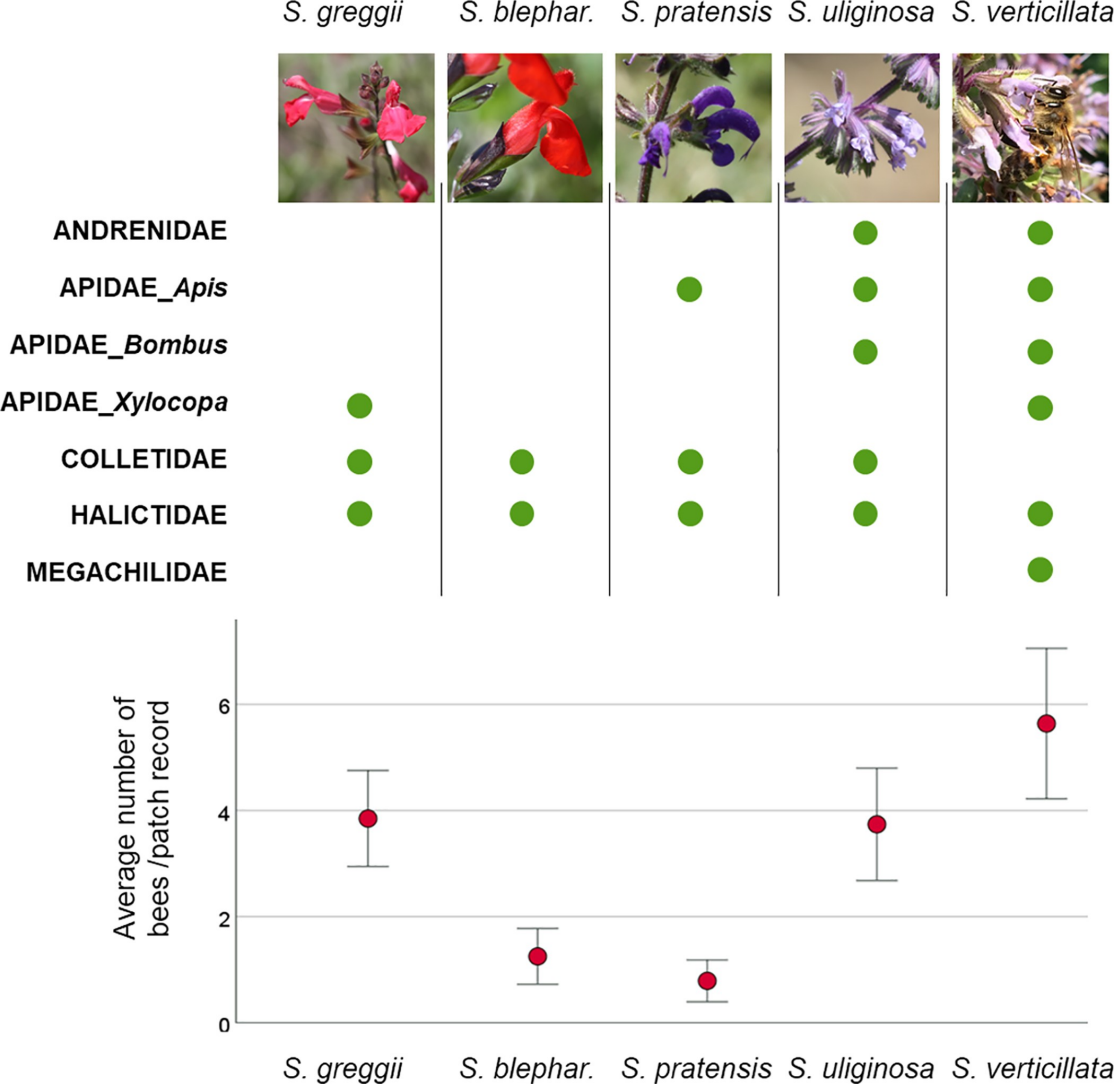
Average number of visits per patch record on the five sages. Upper part of the graph report bee families observed on each *Salvia* species. Bee assemblages (Apidae are split in three subgroups, being the most common and easy to recognise) is addressed by green spots indicating presence on flowers. On the lower graph, error bars are represented with 95% confidence interval. Flowers of each species may be appreciated in the photos on top. In that of *S*. *verticillata*, a honeybee (*Apis mellifera*) is collecting nectar. Photo credits: M. Giovanetti and D. Lupi.

Bees were reported even on *S*. *greggii*, considered an ornitophilous species. For *S*. *blepharophylla*, we could not find any report of direct observations, inside or outside its home range. During our observations, we also recorded the bee families previously listed in the literature (Fig 3, dots refer to presence on flowers). A single family was ubiquitous on all sage species: individuals of the family Halictidae (mostly *Lasioglossum* Curtis spp.) were observed on all the five documented *Salvia*.

Bee assemblages on the five sages differ. The ornitophilous species were the ones attracting a less diverse group of bees. *S*. *pratensis*, a species spontaneously growing in many areas even adjacent to the GBG, was surprisingly poorly visited and even discarded by *Bombus* sp. During a total of 2210 minutes of focal observations, we recorded 883 visits paid to flowers. Many of the 10-mins slot of observations remained without any bee contact: almost the 30% of all time dedicated to observations. Considering all records combined (Fig 3, lower graph), *S*. *verticillata* was the species with the highest number of records: 0.43 visitors/minute, followed by *S*. *uliginosa* (0.35 visitor/minute), and *S*. *greggii* (0.28 visitor/minute). *S*. *blepharophylla* and *S*. *pratensis* had very few visitors (0.09 and 0.07 visitor/minute, respectively). Kruskal-Wallis H test confirmed the difference among sages (χ2(4) = 61.985, *p* = 0.0001).

### GBG as an interactive learning opportunity

The exhibition titled (in Italian): “*Seduzione Repulsione*: *quello che le piante non dicono*” (*Seduction Repulsion–what plants do not say*) displayed ten panels: titles and content of each is reported in S2 Table. The aim was explaining through clear images, drawings and text, the various media of communication employed by plants and the resultant interactions (either positive, negative, or mutualistic) with other organisms. Similarities with human daily life were underlined, reporting them in the panels as well as in the catalogue. For example, the chapter related to colours starts with a citation and the picture of a painting, “In the style of Kairouan– 1914”, of the famous artist Paul Klee. After a trip to Tunisia, the artist finally moved away from black and white works, amazed by colour variability due to intense light. The chapter continues explaining the origin and importance of the colour green in leaves for photosynthesis, and how it is used as background colour for pollinators and seed dispersers to distinguish flowers and fruits. The text gets deeper by introducing the fact that there are differences in how colours result attractive to different organisms (insects, humans, birds, mammals) and what the chemical compounds responsible for different colours are (anthocyanins, carotenoids, flavonoids), in plants and animals as well. Finally, it concludes underlining that differences in perceived colours may also be the result of differences of the surface bearing them. Therefore, as in this example, each panel/chapter of the catalogue refer to features of major importance in the natural world, but relating them with physical and emotional perceptions more often experienced by people.

The catalogue was published in 2016, directly involving the GBG and the non-profit organisation “Rete degli Orti Botanici della Lombardia”. It was assembled through the contribution of A. Ronchi (texts), P. Berera (graphics), under the scientific supervision of G. Fico, while involving numerous collaborators and national stakeholders (Fondazione Cariplo, Regione Lombardia Agricoltura, Ministero dell'Istruzione dell'Università e della Ricerca). The exhibition, still available, already travelled across 11 Italian locations: Botanical Garden Pavia, 6-18/09/2015; Sala Viscontea (Bergamo, 04/10/2015-31/01/2016); Ghirardi Botanical Garden (Toscolano Maderno, Brescia, 14/05-30/06/2016); Headquarters of StelvioPark (Bormio, Sondrio, 15/07-30/09/2016); Brera Botanical Garden (Milano, 12/12/2016-14/01/2017); Castello di Desenzano (Desenzano del Garda, Brescia, 04/03-02/04/2017); Villa Pisani Bolognesi Scalabrin (Vescovana, Padova, 6-25/04/2017); Villa Litta (Lainate, Milano, 30/04-20/05/2017); Natural History Museum (Venezia, 04/11/2017-18/03/2018); Tenuta Villa Quassa (Ispra, Varese, 7-29/o4/2018); JRC, European Union Joint Research Center (Ispra, Varese, 03/05-19/07/2018). The total number of recorded visitors was 51390, in two years; Venice alone attracted more the half of them (28390), with a presence of about 5600 visitors each month and an almost constant increment in the 5 months. The catalogue is currently available at the BGs belonging to the Network of the Botanical Gardens of Lombardy and through specific requests at segreteria@reteortibotanicilombardia.it.

The venues differed between museal institutions and botanic gardens. At botanic gardens, the exhibition encountered a more selected audience searching for rigorous scientific and botanical in-depth-knowledge, but also fascinated by living organisms and the beauty relying on flowers and green leaves. They were finding out themselves the characteristics described in the panels. Museal institutions host in general a wider public, from elderly people to families to school groups (the Network of the Botanical Gardens of Lombardy, in 2017, sum up a total of 39.049 students), ready to walk around and possibly similarly interested to various topics, from nature to history to art. Museums often advertise special exhibition to enlarge the interest of resident public, or inducing distant one to join. This function was successfully taken on by the travelling exhibition during this work. The success of the exhibition resulted in the translation, in a language rich of links with accessible experiences, the achievement of science. Moreover, the added value was that a large part of the achievements presented were resulting from activities run at the same place of exhibitions: museums, botanic gardens, universities. This was well expressed by those that visited the botanic garden with researchers actively observing and recording pollinators. The enthusiastic interest in researcher’s activities, combined with in-situ explanations, seemed to push the interest towards the connection between plants and pollinators.

## Discussion

### GBG as a plant-pollinator network repository

According to Hymenoptera behaviour, we may expect occasional probation in new food sources; repeated visits confirm instead appreciation of resources offered by the plant. Bee visits on exotic flowers have long been recorded, even on invasive species from very distant origin [55]. Visitation rates may develop from occasional visits to the development of a given routine for resource collection [56]. From the results of the present study, we can conclude that the majority of visited plants in the GBG was actively looked for by local pollinators. This indicates that, in absence of co-evolutionary processes that may have built a solid relationship between a plant species and its pollinators [57,58], there are equally attractive forces that favour the establishment of new relationships [59,60]. Similarity of floral morphologies (Fig 1) is certainly the most evident trait possibly justifying this conclusion. However, future data on resource availability may integrate the current findings.

As expected, also pollinators distribution can influence records on visits [61]. Our data pointed a greater abundance of honeybees and bumblebees, when compared to relative abundance of other bee groups. A possible explanation may be linked with honeybee distribution facilitated by men through beekeeping. For the genus *Bombus*, it usually counts on several species when in proximity of mountain areas (i.e. the surroundings of Lake of Garda), where they can find a higher number of suitable nesting sites [62,63].

Generally, we observed that exotic species attracted native pollinators and, depending on species availability and matches with flower characteristics, exotics may even compensate for resources according to flower abundance of native ones. We have to keep in mind that here we did not consider negative effects of possible disruptions of native plants-native pollinators networks, or negative effects due to invasive alien species. A similar result was reported by Lowenstein and colleagues [64], who did not find any effect of biogeographic origin (native versus non-native) of plant species regarding pollinator presence. However, exotic and native flowering ranges at the GBG overlapped and compensated resource offer, with an overall positive effect on local bee assemblages. This is sustained by how linkages (Fig 2) are consistent independently from the origin of a species and is also confirmed by considering the flower shape: in presence of opposite frequencies of head and bilabiate flowers, respectively between exotics and native, we still observe consistency of activity and variety of visiting pollinators. A final consideration deals with future experimental studies on pollen deposition performance and on exotic species habits that may help to interpret linkages as evidenced by Devaux and colleagues [65].

### GBG as an open-air laboratory

Sages are largely renown as ornamentals and/or aromatic species grown in private and common gardens [66]. Notwithstanding, when addressing to their pollinators poor scientific evidence is available. It is worth mentioning that most of the literature we could access refers to pollinator’s observation out of the home range of the target sage (see second column of Table 1). The absence of data on natural conditions turns it difficult to compare native and exotic state of pollinator networks. Global trading of these species and pollinator’s records worldwide, however, are important to formulate the potential plant-pollinator relations to be expected. For example, *S*. *greggii* is definitely attracting the wild large species of the genus *Xylocopa*, in California (USA; literature data) as well as in Italy (Europe). *S*. *pratensis*, interestingly, did not attract even *Bombus* sp., its usual pollinator. This result may eventually be linked to plant location in the garden; however, it may also reflect competition among available resources. Similarly, the large amount of observation set missing bee records could be partially due to adverse weather conditions, or to bees foraging elsewhere on more attractive plant species.

Flower characteristics confirm similarities depending on the pollinators that sages were expected to be coevolved with bees for *S*. *verticillata*, *S*. *pratensis* and *S*. *uliginosa* and birds for *S*. *greggii* and *S*. *blepharophylla*. Dissimilarities in bee attraction among species need to be deeper investigated from an evolutionary point of view, since they were not explained solely by flower traits [67,68]. Among the insect-pollinated ones, *S*. *verticillata* showed an outstanding number of individuals even when compared to *S*. *uliginosa*, the most alike. Similarly, there was a difference between the two ornitophilous species, with *S*. *greggii* accounting for the highest number of bees visiting its flowers. These differences may partly be accounted for flower arrangement on the plant. Sages with medium-sized flowers produces lax inflorescences, while sages with short-sized flowers exhibit dense inflorescences. Flowers are grouped in different types of inflorescences: dense panicle formed by superimposed verticillasters (*S*. *verticillata* and *S*. *uliginosa*), lax spikes with 4–6 flowers in each whorl (*S*. *pratensis*) and lax racemes with 1–2 flowers in each whorl (*S*. *greggii* and *S*. *blepharophylla*). Also, other characteristics may play a role: corolla length and colours. The corolla length varied from the short size class (< 1.5 cm) in *S*. *verticillata* and *S*. *uliginosa* up to the medium size class (1.5–3.0 cm) in the other target species. The corolla colours ranged from the blue tones in *S*. *verticillata* (lilac-blue), *S*. *pratensis* (bluish-violet) and *S*. *uliginosa* (sky-blue with white bee-line on the upper and lower lips) up to the red ones in *S*. *greggii* (scarlet red) and *S*. *blepharophylla* (red with an orange undertone).

Finally, data suggest that the choice of planting these species with the double function of delighting view and sustain local pollinators should favour *S*. *verticillata* and *S*. *greggii*, with different flower traits, colours and pollinator assemblages.

### GBG as an interactive learning opportunity

The Botanic Gardens are places of knowledge, windows on the world of science from which everyone can look out. However, the exhibition contributed a technical and scientific prospective through the development of an awareness-raising process on the importance of the plant biodiversity and how it is linked to the surrounding world. Botanic gardens are very important and worldwide connected by the potential of capacity building in outreach activities and plant rescue and preservation, alive as well as for the respective genetic banks [69,70]. The exhibition received an extraordinary response from the public in terms of both numbers and overall appreciation of the proposed information. The venues differed between museal institutions and botanical gardens. In both cases, the proposal of the traveling exhibition received considerable success. The major strength points are the unusual key of lecture and the language, correct and rigorous from the scientific point of view, but direct and narrative. In the exhibition, we proposed to abandon the most usual man-centred profitable/productive approach to recount the topics related to Repulsion/Attraction through a plant-centred vision. If on the one hand humans were kept out from the vision, on the other human perceptions were used to show plants as living independent organism similar to ourselves. Pollinators played a main role in this view, thanks to the recent worries related to their decrease [27] and the tentative works to define plant lists to contribute to their survival [54,71].

## Conclusions

We aimed at addressing documented public concerns by translating them into research questions to be investigated in a special context, that of a botanical garden. The work arises from citizens’ apprehension described in scientific publications in the field of social sciences, tackled the alarming reports on pollinators losses and dispersion of exotic species, resumed results in outreach activities and material. The first set of data addressed to worries on exotic species planted in private or common gardens, especially on their interactions with the native fauna. Our results highlighted the establishment of similar relationships between exotic or native plants, and local pollinators. Therefore, it can be assumed that (at least some) exotic species may equally contribute to sustain local pollinator fauna thanks to the resource they provide. On this topic, a large amount of literature has offered lists of possible preferred species to be planted when gardening purposes plan intend to match pollinator sustainability [i.e. 22,71]. However, Garbuzov and Ratnieks [50] pointed out a contrasting situation. On the one hand, in existing literature there is generally a low overlap of species even when addressing the same geographic region (and even other pitfalls, as poor recommendations, omitted species, lack of details), finally not providing a decisive list. However, on the other hand, these lists have a strong appeal and could turn them into a communication tool with the potential of driving further research. Our second set of data applied this concept, by choosing renown species used both, for gardening (thanks to their intense and long flowering) and culinary purposes (thanks to their chemical properties). They are very frequently mentioned in gardening articles, printed or online, and used in private as well as public gardens. Our data highlighted that different species of the same genus *Salvia* attracted a different assemblage of pollinators, and therefore a selection among species could be actively performed if their planting is also intended to sustain pollinators.

Botanic gardens are present in most cities, and even in small towns. They mostly host a combination of native and exotic species, and research as well as outreach activities. These two competencies are, however, mostly kept separated. We merged them by transforming the botanic garden in the promoter of widening research activity and outputs on plant-animal interactions, by linking them with physical and emotional human experiences. The exhibition and the catalogue describe how plants interact with other organisms and how we (humans) also use similar modes of interaction: attraction means and repulsion modes coming from similar chemical or mechanical approaches. The exhibition, being a travelling one, has and still is enlarging the audience of this output; the catalogue stands as an inspiring tool.

## Supporting information

S1 Table Actual observations related to plant-pollinator networks at Ghirardi’s Botanic Garden. (DOCX)

S2 Table Description of the panel prepared for the travelling exhibition. The language used on panels was Italian, therefore in the table we kept the original title. However, for a wider understanding explanation of each panel has been translated in English.
(DOCX)

## References

[1] Braito MT, Böck K, Flint C, Muhar A, Muhar S., Penker M. Human-nature relationships and linkages to environmental behaviour. Environ. Values. 2017; 26(3): 365–389.

[2] Tzoulas K, Korpela K, Venn S, Yli-Pelkonen V, Kaźmierczak A, Niemela J, et al Promoting ecosystem and human health in urban areas using Green Infrastructure: A literature review. Landsc Urban Plan. 2007; 81(3): 167–178.

[3] Van Kamp I, Leidelmeijer K, Marsman G, De Hollander A. Urban environmental quality and human well-being: Towards a conceptual framework and demarcation of concepts; a literature study. Landsc Urban Plan. 2003; 65(1–2): 5–18.

[4] Vlek C, Steg L. Human Behavior and Environmental Sustainability: Problems, Driving Forces, and Research Topics. J Soc Issues. 2007, 63(1): 1–19.

[5] Takano T, Nakamura K, Watanabe M. Urban residential environments and senior citizens’ longevity in megacity areas: the importance of walkable green spaces. J Epidemiol Community Health. 2002; 56(12): 913–918. 10.1136/jech.56.12.913 12461111 PMC1756988

[6] Zhang W, Ricketts TH, Kremen C, Carney K, Swinton SM. Ecosystem services and disservices to agriculture. Ecol Econ. 2007; 64(2): 253–260.

[7] Steffan‐Dewenter I, Westphal C. The interplay of pollinator diversity, pollination services and landscape change. J Appl Ecol. 2008; 45(3): 737–741.

[8] Eckert CG, Kalisz S, Geber MA, Sargent R, Elle E, Cheptou PO, et al Plant mating systems in a changing world. Trends Ecol Evol. 2010; 25(1): 35–43. 10.1016/j.tree.2009.06.013 19683360

[9] Barrett SC, Harder LD. The ecology of mating and its evolutionary consequences in seed plants. Annu Rev Ecol Evol Syst. 2017; 48: 135–157.

[10] Vanbergen AJ, Woodcock BA, Heard MS, Chapman DS. Network size, structure and mutualism dependence affect the propensity for plant–pollinator extinction cascades. Funct Ecol, 2017; 31(6): 1285–1293.

[11] Vanbergen AJ, Initiative TIP. Threats to an ecosystem service: pressures on pollinators. Front Ecol Environ. 2013; 11(5): 251–259.

[12] Kremen C, Williams NM, Thorp RW. Crop pollination from native bees at risk from agricultural intensification. Proc Natl Acad Sci. 2002; 99(26): 16812–16816. 10.1073/pnas.262413599 12486221 PMC139226

[13] Murray TE, Kuhlmann M, Potts SG. Conservation ecology of bees: populations, species and communities. Apidologie. 2009; 40(3): 211–236.

[14] Hoyle H, Hitchmough J, Jorgensen A. Attractive, climate-adapted and sustainable? Public perception of non-native planting in the designed urban landscape. Landsc Urban Plan. 2017; 164: 49–63.

[15] Jabareen Y (2013). Planning the resilient city: Concepts and strategies for coping with climate change and environmental risk. Cities, 31: 220–229.

[16] Swanwick C, Dunnett N, Woolley H. Nature, role and value of green space in towns and cities: An overview. Built Environ. 2003: 94–106.

[17] Baró F, Haase D, Gómez-Baggethun E, Frantzeskaki N. Mismatches between ecosystem services supply and demand in urban areas: A quantitative assessment in five European cities. Ecol indic. 2015; 55: 146–158.

[18] Bolund P, Hunhammar S. Ecosystem services in urban areas. Ecol econ. 1999; 29(2): 293–301.

[19] Frankie G, Thorp R, Hernandez J, Rizzardi M, Ertter B, Pawelek J, et al Native bees are a rich natural resource in urban California gardens. Calif Agric. 2009; 63(3): 113–120.

[20] Pawelek JC, Frankie GW, Thorp RW, Przybylski M. Modification of a community garden to attract native bee pollinators in urban San Luis Obispo, California. Cities and Environ. 2009; 2(1): 1–20.

[21] Burr A, Hall DM, Schaeg N. The perfect lawn: exploring neighborhood socio-cultural drivers for insect pollinator habitat. Urban Ecosyst. 2018; 21(6): 1123–1137.

[22] Hicks DM, Ouvrard P, Baldock KC, Baude M, Goddard MA, Kunin WE., et al Food for pollinators: quantifying the nectar and pollen resources of urban flower meadows. PloSone. 2016; 11(6): e0158117. 10.1371/journal.pone.0158117 PMC4920406 27341588

[23] Lowenstein DM, Matteson KC, Minor ES. Evaluating the dependence of urban pollinators on ornamental, non-native, and ‘weedy’floral resources. Urban Ecosyst. 2019; 22: 293–302.

[24] Schlaepfer MA, Sax DF, Olden JD. The potential conservation value of non‐native species. Conserv Biol. 2011; 25(3): 428–437. 10.1111/j.1523-1739.2010.01646.x 21342267

[25] Lozier JD, Zayed A. Bee conservation in the age of genomics. Conserv Genet. 2017; 18(3): 713–729.

[26] Neumann P, Carreck NL. Honey bee colony losses. J Apic Res. 2010; 49 (1): 60–65.

[27] Potts SG, Biesmeijer JC, Kremen C, Neumann P, Schweiger O, Kunin WE. Global pollinator declines: trends, impacts and drivers. Trends Ecol Evol. 2010; 25(6): 345–353. 10.1016/j.tree.2010.01.007 20188434

[28] Ramankutty N, Mehrabi Z, Waha K, Jarvis L, Kremen C, Herrero M, et al Trends in global agricultural land use: implications for environmental health and food security. Ann Rev Plant Biol. 2018; 69: 789–815. 29489395 10.1146/annurev-arplant-042817-040256

[29] Schönfelder ML, Bogner FX. Individual perception of bees: Between perceived danger and willingness to protect. PloSone. 2017; 12(6), e0180168. 10.1371/journal.pone.0180168 PMC5491143 28662124

[30] Sanders DL, Ryken AE, Stewart K. Navigating nature, culture and education in contemporary botanic gardens. Environ Educ Res. 2018; 24(8): 1077–1084.

[31] Giovanetti M, Cervera JC, Andrade JL. Pollinators of an endemic and endangered species, *Mammillaria gaumeri* (Cactaceae), in its natural habitat (coastal dune) and in a botanical garden. Madroño. 2007; 54(4): 286–292.

[32] Gaio-Oliveira G, Delicado A, Martins-Loução MA. Botanic gardens as communicators of plant diversity and conservation. Bot Rev. 2017; 83(3): 282–302.

[33] Clauser M, Pavone P. Orti Botanici. Eccellenze Italiane. Thema Edizioni, Città di Castello (Perugia). 2016.

[34] Jackson PW, Kennedy K. The global strategy for plant conservation: a challenge and opportunity for the international community. Trends Plant Sci. 2009; 4(11): 578–580. 10.1016/j.tplants.2009.08.011 19781974

[35] Mounce R, Smith P, Brockington S. *Ex situ* conservation of plant diversity in the world’s botanic gardens. Nature Plants. 2017; 3(10): 795–802. 10.1038/s41477-017-0019-3 28947807

[36] Waylen K. Botanic gardens: using biodiversity to improve human wellbeing. Med Plant Cons. 2006; 12: 4–8.

[37] Burgess J, Harrison CM, Limb M. People, parks and the urban green: a study of popular meanings and values for open spaces in the city. Urban Stud. 1988; 25(6): 455–473.

[38] Chiesura A. The role of urban parks for the sustainable city. Landsc Urban Plan. 2004; 68(1): 129–138.

[39] Church SP. From street trees to natural areas: retrofitting cities for human connectedness to nature. J Environ Plan Manag. 2018; 61(5–6): 878–903.

[40] Kareiva P, Watts S, McDonald R, Boucher T. Domesticated nature: shaping landscapes and ecosystems for human welfare. Science. 2007; 316(5833): 1866–1869. 10.1126/science.1140170 17600209

[41] Berera P, Ronchi A, Fico G. Seduzione repulsione quello che le piante non dicono. Grafo, Palazzago; Italy; 2016.

[42] Soldati M, Marchetti M (ed.). Landscapes and landforms of Italy. Springer, 2017.

[43] Colombo L. Oriente in Orto Botanico–East in the Botanic Garden. Master Thesis in Herbal Sciences and Technologies, University of Milan, Milan, Academic year 2017–2018. 2018.

[44] Pellissier L, Pottier J, Vittoz P, Dubuis A, Guisan A. Spatial pattern of floral morphology: possible insight into the effects of pollinators on plant distributions. Oikos. 2010; 119(11): 1805–1813.

[45] Giuliani C, Ascrizzi R, Tani C., Bottoni, Maleci Bini LM, Flamini G, et al *Salvia uliginosa* Benth.: glandular trichomes as bio-factories of volatiles and essential oil. Flora. 2017°; 233: 12–21.

[46] Giuliani C, Ascrizzi R, Corrà S, Maleci Bini L, Flamini G, Fico G. Ultrastructural insight into terpene-producing trichomes and essential oil profile in *Salvia greggii* A. Gray. Flora. 2017b; 236: 107–114.

[47] Giuliani C, Ascrizzi R, Lupi D, Tassera G, Santagostini L, Giovanetti M, et al *Salvia verticillata*: Linking glandular trichomes, volatiles and pollinators. Phytochemistry. 2018; 155: 53–60. 10.1016/j.phytochem.2018.07.016 30077120

[48] Waites AR., Ågren JON. Pollinator visitation, stigmatic pollen loads and among‐population variation in seed set in *Lythrum salicaria*. J Ecol. 2004; 92(3): 512–526.

[49] Wester P, Claßen-Bockhoff R. Floral diversity and pollen transfer mechanisms in bird-pollinated *Salvia* species. Ann Botany. 2007; 100(2): 401–421. 17522077 10.1093/aob/mcm036 PMC2735312

[50] Garbuzov M, Ratnieks FLW. Listmania: The Strengths and Weaknesses of Lists of Garden Plants to Help Pollinators, BioScience. 2014; 64(11): 1019–1026. ttps://doi.org/10.1093/biosci/biu150

[51] Bozek M. Flowering biology and pollen flow of three species from genus *Salvia* L. (in Polish) Annales Universitatis Mariae Curie-Sklodowska. SectioN EEE Horticultura. 2002; 10: 51–57

[52] Teper D. Food plants of *Bombus terrestris* L. determined by palynological analysis of pollen loads. J Apic Science. 2004; 48(2): 75–81.

[53] Toopchi-Khosroshahi Z, Lotfalizadeh HA. Identification of honey plants and their attractiveness to honeybee in Kandovan, Northwest of Iran. Biharean Biol. 2011; 5(1): 36–41.

[54] Corbet SA, Bee J, Dasmahapatra K, Gale S, Gorringe E, La Ferla B, et al Native or exotic? Double or single? Evaluating plants for pollinator-friendly gardens. Ann Botany. 2001; 87(2): 219–232. 10.1006/anbo.2000.1322 32050738

[55] Giuliani C, Giovanetti M, Foggi B, Mariotti Lippi M. Two alien invasive acacias in Italy: differences and similarities in their flowering and insect visitors. Plant Biosyst. 2016; 150(2): 285–294.

[56] Giovanetti M, Mariotti Lippi M, Foggi B, Giuliani C. Exploitation of the invasive Acacia pycnantha pollen and nectar resources by the native bee *Apis mellifera*. Ecol Res. 2015; 30(6): 1065–1072.

[57] Van der Niet T, Peakall R, Johnson SD. Pollinator-driven ecological speciation in plants: new evidence and future perspectives. Ann Botany. 2014; 113(2): 199–212. 24418954 10.1093/aob/mct290 PMC3890394

[58] Gómez JM, Perfectti F, Lorite J. The role of pollinators in floral diversification in a clade of generalist flowers. Evolution. 2015; 69(4): 863–878. 10.1111/evo.12632 25757195

[59] McKinney AM, Goodell K. Plant–pollinator interactions between an invasive and native plant vary between sites with different flowering phenology. Plant Ecol. 2011; 212(6): 1025–1035.

[60] Kaiser-Bunbury CN, Müller CB. Indirect interactions between invasive and native plants via pollinators. Naturwissenschaften. 2009; 96(3): 339–346. 10.1007/s00114-008-0481-x 19050842

[61] Rodríguez-Gironés MA, Santamaría L. Models of optimal foraging and resource partitioning: deep corollas for long tongues. Behav Ecol. 2006; 17(6): 905–910.

[62] Kells AR, Goulson D. Preferred nesting sites of bumblebee queens (Hymenoptera: Apidae) in agroecosystems in the UK. Biol cons. 2003 109(2): 165–174.

[63] Ruffo S, Stoch F (eds). Checklist e distribuzione della fauna italiana. Memorie del Museo. Civico di Storia Naturale di Verona, 2 serie, Sezione Scienze della Vita 16. 2005

[64] Lowenstein DM, Matteson KC, Minor ES. Evaluating the dependence of urban pollinators on ornamental, non-native, and ‘weedy’floral resources. Urban Ecosyst. 2019; 22(2): 293–302.

[65] Devaux C, Lepers C, Porcher E. Constraints imposed by pollinator behaviour on the ecology and evolution of plant mating systems. J Evol Biol. 2014; 27(7): 1413–1430. 10.1111/jeb.12380 24750302

[66] Betsy C. A book of salvias: sages for every garden. Timber Press, Incorporated, Oregon 1997

[67] Hu SS, Dilcher DL, Jarzen DM& Taylor DW. Early steps of angiosperm-pollinator coevolution. Proc Natl Acad Sci USA. 2008; 105: 240–245. 10.1073/pnas.0707989105 18172206 PMC2224194

[68] Rosas-Guerrero V, Aguilar R, Martén-Rodríguez S, Ashworth L, Lopezaraiza-Mikel M, Bastida JM, et al A quantitative review of pollination syndromes: do floral traits predict effective pollinators? Ecol letters. 2014; 17(3): 388–400. 10.1111/ele.12224 24393294

[69] Heywood VH, Jackson PW (Eds.). Tropical botanic gardens: their role in conservation and development. Academic Press (2012).

[70] Sharrock S, Chavez M. The role of Botanic Gardens in building capacity for plant conservation. BGjournal, 2013; 10(1), 3–7.

[71] Salisbury A, Armitage J, Bostock H, Perry J, Tatchell M, Thompson K. Enhancing gardens as habitats for flower‐visiting aerial insects (pollinators): should we plant native or exotic species? J App Ecol. 2015; 52(5): 1156–1164.

