# Supplementary material for: A botanic garden as a tool to combine public perception of nature and life-science investigations on native/exotic plants interactions with local pollinators
Source: PLoS One. 2020 Feb 20;15(2):e0228965. doi: 10.1371/journal.pone.0228965 (PMC7032708; doi:10.1371/journal.pone.0228965)
Supplement: S1 Table — (DOCX) [file pone.0228965.s001.docx]

S1 Table: Actual observations related to plant-pollinator networks at Ghirardi’s Botanic Garden

| Plants in the Ghirardi’s Botanic Garden | | | | | | | | | | | |
| --- | --- | --- | --- | --- | --- | --- | --- | --- | --- | --- | --- |
| Species present | | | Species visited | | | | | Species visited | | | |
|  |  |  |  |  |  |  |  | occasionally | | | repeatedly |
| Total number of species* | 244 | | Total visited species* | | | 140 | | 49 | | | 91 |
| Total exotic species* | 189 | | Total exotic visited species* | | | 62 | | 26 | | | 36 |
| Total native species* | 310 | | Total native visited species* | | | 78 | | 23 | | | 55 |
|  | | | | | | | | | | | |
|  | | | Number of visited species per flower morphotype | | | | Number of visits per flower morphotype | | | | |
|  |  |  | exotic | | native | | exotic | | | native | |
| Bilabiate | | | 12 | | 23 | | 32 | | | 122 | |
| Disk | | | 19 | | 18 | | 50 | | | 54 | |
| Funnel | | | 20 | | 24 | | 49 | | | 101 | |
| Head | | | 8 | | 12 | | 54 | | | 32 | |
| Tube | | | 1 | | 0 | | 2 | | | 0 | |
| Wind | | | 2 | | 1 | | 2 | | | 1 | |
| * recorded along the transects; in the entire garden, more than 400 taxa are represented.  The four *Salvia* sp. included in following analyses are excluded from this list. | | | | | | | | | | | |
| Bee visits in the Ghirardi’s Botanic Garden | | | | | | | | | | | |
| Total number of visits^§^ | | 517 | | Total number of bee families | | | | | 5 | | |
| Total records of Andrenidae | | 14 | | Total records of Halictidae | | | | | 125 | | |
| Total records of Apidae | | 281 | | Total records of Megachilidae | | | | | 50 | | |
| Total records of Colletidae | | 27 | |  | | | | |  | | |
| ^§^including unascertained data | | | | | | | | | | | |
