# Supplementary material for: A botanic garden as a tool to combine public perception of nature and life-science investigations on native/exotic plants interactions with local pollinators
Source: PLoS One. 2020 Feb 20;15(2):e0228965. doi: 10.1371/journal.pone.0228965 (PMC7032708; doi:10.1371/journal.pone.0228965)
Supplement: S2 Table — The language used on panels was Italian, therefore in the table we kept the original title. However, for a wider understanding explanation of each panel has been translated in English. (DOCX) [file pone.0228965.s002.docx]

S2 Table: Description of the panel prepared for the travelling exhibition. The language used on panels was Italian, therefore in the table we kept the original title. However, for a wider understanding explanation of each panel has been translated in English.

| ***Description of exhibition panel content*** |
| --- |
| 1. FORME - *Shapes*  Both at microscopic and macroscopic scale, plants have evolved “*endless forms most beautiful and most wonderful*” (*Charles Darwin – The Origin of Species, 1859*) to adapt to the environment, to attract pollinators or to repel predators. Ingenious and sophisticated solutions to guarantee the perpetuation of the species |
| 2. COLORI - *Colours*  The colours of flowers and fruits enchant, attract, seduce. Not only pollinators or seed dispersers, but also humans. Humans however can only partially appreciate the seductive value of flowers and fruits. |
| 3. ODORI - *Smells*  Smells are signals emitted by plants to attract or repel even those who are far away. The olfactory memory contains the promise of a reward or the warning of a potential danger. |
| 4. SAPORI – *Flavours*  Plant offer “parts of themselves” through the production of attractive flavours to ensure the reproduction success. On the contrary, they produce unpleasant flavours to repel predators. |
| 5. VELENI - *Poisons*  Plants produce poisons for their own defence: sometimes the whole plant is poisonous, sometimes just some parts. However, it is the dose that makes the poison. Animals know this well, so that they completely avoid the plant or use it in a conscious way. |
| 6. INGANNO - *Deception*  As in the myths, even in the plant kingdom the seductive strategies hiding a deception are frequent: sometimes deception can be accompanied by a reward, but it is not always the case. Victims are often insects or small animals that are attracted by shapes, colours or scents. |
| 7. COOPERAZIONE - *Cooperation*  Plants are anything that voiceless. They emit a plethora of chemical signals, which, just like shouts, can discourage potential aggressors, but also call on rescuers and warn other plants to face the imminent danger. |
| 8. SEDUZIONE DEL TRAMONTO – *Seduction of the sunset*  Leaves fall, flowers wither but this is not the end. When a plant part dies, new energy becomes available for the development of other structures, in a cyclical renewal in which nothing dies forever. |
| 9. TUTTO E' RELATIVO – *Everything is relative*  Beautiful and useful plants for humans can become harmful for the conservation of natural ecosystems. Indeed, animal or plant species, voluntarily introduced in the past for ornamental or production purposes, can severely threaten the local biodiversity, due to their massive spreading in the wild. |
| 10. RICERCA SCIENTIFICA – *Scientific research*  Plants produce our food, timber, fuel, clothes, paper, medicines, colours, oxygen. Plants feed the planet and make it breathe. Our life on earth depends on plants. The future of our agriculture and economy depend on the new ideas of scientists who study plants. The research concerns us. |
